# Which Factors Predict 30-Day Readmission After Total Hip and Knee Replacement Surgery?

**DOI:** 10.7759/cureus.23093

**Published:** 2022-03-12

**Authors:** Cynthia L Williams, George Pujalte, Zhuo Li, Rock P Vomer, Maruoka Nishi, Lisa Kieneker, Cedric J Ortiguera

**Affiliations:** 1 Therapeutics, Brooks College of Health, University of North Florida, Jacksonville, USA; 2 Department of Family Medicine and Department of Orthopedics, Mayo Clinic, Jacksonville, USA; 3 Biostatistics Unit, Mayo Clinic, Jacksonville, USA; 4 Family Medicine, Eastern Virginia Medical School, Norfolk, USA; 5 Department of Family and Community Medicine, University of Oklahoma, Tulsa, USA; 6 Family Medicine, Mayo Clinic, Jacksonville, USA; 7 Orthopedic Surgery, Mayo Clinic, Jacksonville, USA

**Keywords:** surgeries, readmissions, procedures, orthopedic, arthroplasty

## Abstract

Background

The Centers for Medicare and Medicaid Services enacted the Hospital Readmissions Reduction Program to impose penalties for diagnoses with high readmission rates. Despite several elective orthopedic procedures being included in this program, readmission rates have not declined, and associated costs have reached critical levels for total knee and total hip arthroplasty. Readmissions drastically impact patient outcomes. There are many known contributors to patient readmission rates, including infection, pain, and hematomas. However, evidence is inconclusive regarding other aspects, such as demographics, insurance, and discharge disposition. The purpose of this manuscript is to 1) measure hospital readmission rates for total knee and total hip arthroplasty, 2) evaluate the causes of readmissions, and 3) provide a predictive profile of risk factors associated with hospital readmissions.

Methods

Patients who underwent total knee or total hip arthroplasty were identified through a retrospective database review. An electronic chart review extracted data concerning patient demographics, comorbidities, surgical information, 30-day outcomes, and reasons for 30-day readmissions. Continuous and categorical variables were assessed with the Wilcoxon rank-sum test and the Chi-square test, respectively.

Results

A total of 6,065 patients were included, with 269 (4.4%) having at least one surgery-related 30-day readmission. No differences in readmission were noted with age, sex, or ethnicity; however, differences were found in weight and body mass index. Statistically significant comorbidities were heart failure, chronic obstructive pulmonary disease, dialysis, and alcohol use or abuse.

Conclusion

Our research indicated that surgery type, length of stay, and heart failure most significantly impacted 30-day readmission rates. By assessing readmission rates, we can take steps to optimize care for non-elective surgeries that will improve patient outcomes and cost-effectiveness.

## Introduction

Hospital 30-day readmission rates (i.e., hospital admissions that occur within 30 days of initial discharge, excluding patients who were in the hospital for less than 24 hours) have increased to fiscally unsustainable proportions. Given this problem, in 2012, the Centers for Medicare and Medicaid Services enacted the Hospital Readmissions Reduction Program to impose payment penalties for diagnoses with high hospital readmission rates. In 2014, this list of diagnoses was expanded to include orthopedic surgeries, such as total knee arthroplasty (TKA) and total hip arthroplasty (THA). However, the expansion of the Hospital Readmissions Reduction Program has not led to great declines in readmissions [1-4].

Unplanned 30-day hospital readmissions for total joint replacements have reached critical numbers; they account for 55% of unplanned orthopedic readmissions, costing an average of approximately $38,953 for TKA and $36,038 for THA per readmission and contributing $18.75 billion annually to Medicare expenditures [5,6]. Thirty-day readmission rates are 5.56% and 3.21% for inpatient TKA and THA, respectively, and 4% and 2.95% for outpatient TKA and THA, respectively [7]. This significantly affects health care costs, as a 1% increase in hospital readmission rates is associated with an average 1.2% increase in non-reimbursable hospital expenses [5].

In light of similar studies, it can be reasonably concluded that physicians may affect changes to quality outcomes when they understand how patient characteristics, comorbidities, and decision-making influence readmissions. Hospital readmission rates for TKA and THA considerably influence patient outcomes and hospital quality. Studies have shown that surgical site infections, pain, and hematomas are significant contributors to readmissions, as is discharge disposition, which refers to the place of postoperative care, usually a skilled nursing facility or the patient’s home, where home health care is provided [6-9]. Bernatz and colleagues suggest that patients admitted to a skilled nursing facility after surgery are more likely to be readmitted to the hospital than those discharged to their homes with home health care [10].

However, other studies in the literature are inconclusive about the influence of insurance, discharge disposition, and length of stay (LOS) on hospital readmissions [9-12]. Some researchers speculate that the shorter the hospital stay, the more likely hospital readmission [9,10,13]. Basques et al. have found that men are more likely than women to suffer adverse events and are at an increased risk of hospital readmission [14]. Similarly, Black American patients, patients with lower incomes, treated at lower-volume hospitals or insured by Medicare or Medicaid, and patients who have reduced access to hospitals (i.e., because they live longer distances away) are more likely to experience increased readmission rates [7,15].

As demand and costs continue to increase for TKA and THA, readmissions have become an enormous concern for health care institutions, which seek to provide high-quality, value-based care while reducing costs. As the population ages and obesity rates continue to rise in the US, the demand for TKA and THA is expected to surpass four million cases by 2030 [16]. Therefore, developing a comprehensive understanding of the most at-risk populations is critical for effectively using resources to reduce hospital readmission rates and mitigate costs. Such knowledge of this issue will allow providers to develop a predictive profile, which can be used to target resources more effectively, lessening readmissions for the most at-risk patients. Therefore, the purpose of this research is to provide a profile of the patients who are most at risk for unexpected 30-day hospital readmissions. The study’s objectives are to 1) measure hospital readmission rates for TKA and THA, 2) evaluate the causes of readmissions, and 3) provide a predictive profile of risk factors associated with hospital readmissions.

## Materials and methods

A retrospective administrative database review was performed to identify patients who had undergone TKA or THA at Mayo Clinic between January 1, 2013, and December 31, 2018. This study was approved by the Mayo Clinic Institutional Review Board. Data were de-identified and stored in a secure Health Insurance Portability and Accountability Act-compliant database. Patients were identified by current procedural terminology for TKA, revision TKA, THA, and revision THA. Only unique patients aged 18 or older were included, such that the first episode per patient during the study period was included to mitigate selection bias caused by patients with multiple hospital readmissions for qualifying procedures. Patient demographics, baseline comorbidities, surgical information, 30-day outcomes, and reasons for 30-day readmission were retrieved from the electronic chart review. Patients who received non-elective surgery are defined TKA/THAs for fractures/trauma; elective surgeries are TKA/THA due to osteoarthritis. BMI values were classified as follows: underweight, 10<BMI<18.5; normal, 18.5≤BMI<25; overweight, 25≤BMI<30; and obese, 30≤BMI<50.

Continuous variables were summarized as mean (SD) and median (range), while categorical variables were reported as frequency (percentage). Continuous variables were compared between patients with and without 30-day readmission via the Wilcoxon rank-sum test, and categorical variables were compared using the Chi-square (χ2 ) test.

To fit and validate the prediction model, the whole cohort was randomly split into a training set and a validation set at an 8:2 ratio. The random forest method was used to select variables of importance in the training set. A multivariable logistic regression model was fit based on the most important variables that also showed statistical significance. Validation was conducted by applying the model estimate to the validation set and calculating the area under the curve. All tests were two-sided, and R 3.6.2 statistical software (R Foundation for Statistical Computing, Vienna, Austria) was used for data analysis.

Missing data occurred for a few predictors, including ethnicity, body mass index (BMI), smoking status, and alcohol usage. When smoking or alcohol usage was not mentioned, we assumed patients did not use them. For ethnicity and BMI, a complete case analysis was used.

## Results

After chart reviews and the removal of duplicate patients, 6,065 patients were included in the analysis. As noted in Tables 1-2, the median (range) age was 68 (18-101) years, and 2.571 (42.4%) were men while 3.494 (57.6%) were women.

**Table 1:** Patient characteristics and demographics BMI - body mass index ^a^ p-values arise from either a Kruskal-Wallis or a χ2 goodness-of-fit test

Characteristic	No 30-day readmission (n=5,796)	At least 1 30-day readmission (n=269)	Total (N=6,065)	P-value^a^
Age at surgery, years				0.20
Mean (SD)	67.4 (13.2)	68.6 (14.6)	67.4 (13.3)	
Median (range)	68.2 (18.2-101.1)	68.8 (20.0-98.5)	68.2 (18.2-101.1)	
Sex, n (%)				0.45
Male	2,451 (42.3)	120 (44.6)	2,571 (42.4)	
Female	3,345 (57.7)	149 (55.4)	3,494 (57.6)	
Ethnicity, n (%)				0.91
Missing	167	9	176	
Hispanic or Latino	180 (3.2)	8 (3.1)	188 (3.2)	
Not Hispanic or Latino	5,449 (96.8)	252 (96.9)	5,701 (96.8)	
Height, cm				0.16
Missing	250	17	267	
Mean (SD)	168.9 (11.2)	167.6 (10.6)	168.8 (11.2)	
Median (range)	168.0 (123.0-463.0)	167.0 (126.4-191.0)	168.0 (123.0-463.0)	
Weight, kg				0.003
Missing	225	16	241	
Mean (SD)	86.6 (25.5)	82.6 (23.5)	86.4 (25.4)	
Median (range)	84.8 (31.8-780.0)	80.0 (38.8-173.8)	84.7 (31.8-780.0)	
BMI, n (%)				<0.001>
Missing	304	20	324	
Normal	1,141 (20.8)	60 (24.1)	1,201 (20.9)	
Underweight	65 (1.2)	11 (4.4)	76 (1.3)	
Overweight	1,793 (32.6)	82 (32.9)	1,875 (32.7)	
Obese	2493 (45.4)	96 (38.6)	2,589 (45.1)	

**Table 2:** Comorbidities COPD - chronic obstructive pulmonary disease ^a^ p-values arise from either a Kruskal-Wallis or a χ2 goodness-of-fit test

Comorbidity	No 30-day readmission (n=5,796)	At least 1 30-day readmission (n=269)	Total (N=6,065)	P-value^a^
Diabetes				0.14
No	4,931 (85.1)	220 (81.8)	5,151 (84.9)	
Yes	865 (14.9)	49 (18.2)	914 (15.1)	
Heart failure				<0.001>
No	5,367 (92.6)	223 (82.9)	5,590 (92.2)	
Yes	429 (7.4)	46 (17.1)	475 (7.8)	
COPD				0.03
No	5,178 (89.3)	229 (85.1)	5,407 (89.2)	
Yes	618 (10.7)	40 (14.9)	658 (10.8)	
Dialysis				<0.001>
No	5,774 (99.6)	263 (97.8)	6,037 (99.5)	
Yes	22 (0.4)	6 (2.2)	28 (0.5)	
Smoking status				0.06
Current or former	2,585 (44.6)	104 (38.7)	2,689 (44.3)	
Never	3,211 (55.4)	165 (61.3)	3,376 (55.7)	
Have you had alcohol abuse?				0.04
No	5,700 (98.3)	260 (96.7)	5,960 (98.3)	
Yes	96 (1.7)	9 (3.3)	105 (1.7)	

Two hundred sixty-nine (4.4%) patients had at least one surgery-related 30-day readmission. The comparison of demographics, comorbidities, and surgical variables between patients with and without readmission showed that there were no notable differences for at least one surgery-related 30-day readmission in age, sex, ethnicity, or height. However, significant differences were noted in risk factors such as weight with a mean of 82.6±23.5 for readmitted patients vs. 86.6±25.5 for not readmitted patients (p<0.003). Underweight patients were much more likely to have readmission (14.5%) compared to patients in other categories (normal 5%, overweight 4.4%, and obese 3.7%, p<0.001, Table 1). Statistically significant differences were also observed for heart failure (HF): readmission rate was 9.7% with heart failure vs. 4% without HF (p<0.001), chronic obstructive pulmonary disease (COPD): 6.1% with COPD vs. 4.2% without COPD (p=0.03), dialysis: 21.4% with dialysis vs. 4.4% without dialysis (p<0.001), and alcohol use or abuse: 8.6% with alcohol abuse vs. 3.7% without alcohol abuse (p=0.04), see Table 2.

**Figure 1:**
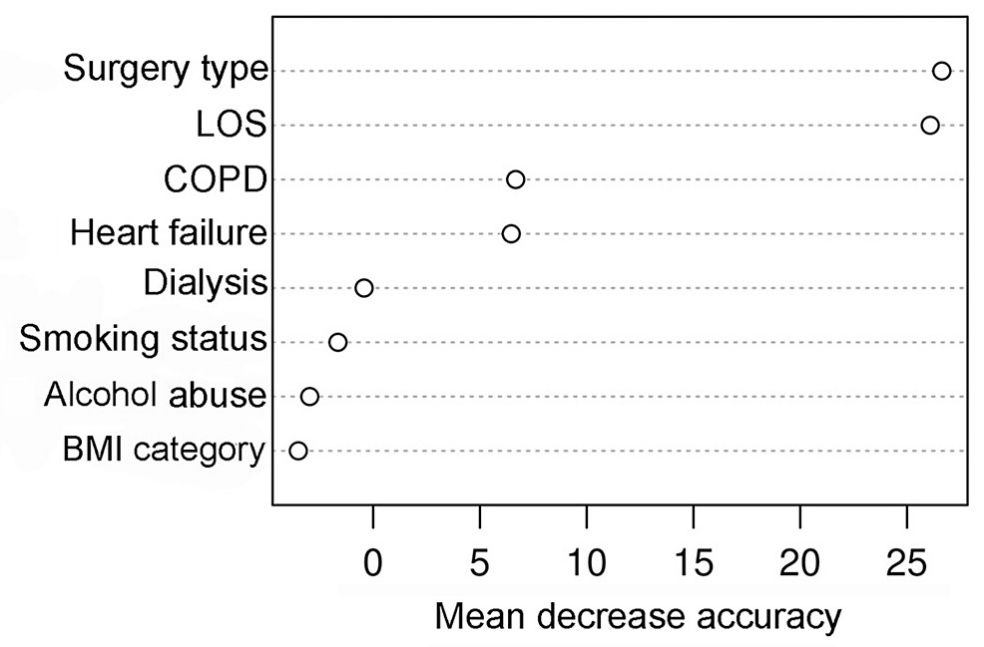
Variables of importance for readmission BMI - body mass index; COPD - chronic obstructive pulmonary disease; LOS - length of stay

Of those readmitted, the main causes for readmission were orthopedic etiology, with 183 events (68%). Statistical significance in LOS was noted for patients with and without readmission (mean 3.1±1.9 vs. 2.6±1.7 days, p<0.001), see Table 3. Etiologic causes for first and second 30-day readmission, respectively, were mainly orthopedic (68% and 50%), medical (13.4% and 16.7%), and gastrointestinal (5.6% and 16.7%) as shown in Tables 4, and the variables of importance, as determined by random forest method, are listed in Table 5. The means decrease accuracy were 26.6 for surgery type, 26.6 for LOS, 6.7 for COPD, and 6.5 for heart failure (see Table 5, Figure 1). Table 6 presents the results of the final multivariable logistic regression model. Surgery type, LOS, and heart failure made the most significant impact on 30-day readmission outcomes. The area under the curve of the model was 0.69, indicating moderate predictive power.

**Table 3:** Surgery summary ^a^ p-values arise from either a Kruskal-Wallis or a χ2 goodness of fit test

Surgical variable	No 30-day readmission (n=5,796)	At least 1 30-day readmission (n=269)	Total (N=6,065)	P-value^a^
Type, n (%)				<0.001>
Elective	5,145 (88.8)	195 (72.5%)	5,340 (88.0)	
Emergent	651 (11.2)	74 (27.)	725 (12.0)	
Length of stay, days				<0.001>
Mean (SD)	2.6 (1.7)	3.1 (1.9)	2.6 (1.7)	
Median (range)	2.0 (0.0-46.0)	3.0 (0.0-13.0)	2.0 (0.0-46.0)	
Year, n (%)				0.49
2013	1,067 (18.4)	48 (17.8)	1,115 (18.4)	
2014	965 (16.6)	53 (19.7)	1,018 (16.8)	
2015	933 (16.1)	42 (15.6)	975 (16.1)	
2016	877 (15.1)	40 (14.9)	917 (15.1)	
2017	975 (16.8)	35 (13.0)	1,010 (16.7)	
2018	979 (16.9)	51 (19.0)	1,030 (17.0)	

**Table 4:** Thirty-day readmissions (n=269) LOS - length of stay

Variable	Total
Number of readmissions within 30 days, n (%)	
1	251 (93.3)
2	17 (6.3)
3	1 (0.4)
First 30-day readmission	
Category, n (%)	
Unknown	1 (0.4)
Cardiac	3 (1.1)
Gastrological	2 (0.7)
Gastrointestinal	15 (5.6)
Hematologic	1 (0.4)
Medical	36 (13.4)
Neurologic	6 (2.2)
Orthopedic	183 (68.0)
Pulmonological	20 (7.4)
Renal	1 (0.4)
Surgical	1 (0.4)
LOS, days	
Mean (SD)	3.5 (3.9)
Median (range)	3.0 (0.0-28.0)
Second 30-day readmission (n=18)	
Category, n (%)	
Gastrointestinal	3 (16.7)
Hematologic	1 (5.6)
Medical	3 (16.7)
Orthopedic	9 (50.0)
Pulmonological	1 (5.6)
Renal	1 (5.6)
LOS, days	
Mean (SD)	3.1 (2.1)
Median (range)	3.0 (0.0-7.0)

**Table 5:** Variable of importance by the random forest method BMI - body mass index; COPD - chronic obstructive pulmonary disease; LOS - length of stay

Variable	Mean decrease accuracy
BMI category	−3.5
Heart failure	6.5
COPD	6.7
Dialysis	−0.4
Surgery type	26.6
LOS	26.1
Smoking status	−1.6
Alcohol abuse	−3.0

**Table 6:** Logistic regression model based on the most important variables (AUC=0.69) AUC - area under the curve; LOS - length of stay; OR - odds ratio ^a^ p-values arise from either a Kruskal-Wallis or a χ2 goodness-of-fit test

Term	OR (95% CI)	P-value^a^
Surgery type, emergent	2.74 (1.87-3.96)	<0.001>
LOS	1.06 (1.00-1.12)	0.02
Heart failure, yes	1.86 (1.22-2.76)	0.003

The predicted probability of 30-day readmission was calculated in the validation set based on the same predictors and the model coefficients estimated from the logistic regression model in the training set. The calculated probability ranged from 0.03 to 0.2. Patients were divided into the following groups: 1) predicted risk of 0.032 or less; 2) predicted risk greater than 0.032, but not greater than 0.033; 3) predicted risk greater than 0.033, but not greater than 0.050; 4) predicted risk greater than 0.050, but not greater than 0.100; and 5) predicted risk greater than 0.100, but not greater than 0.200. The readmission rate revealed an increasing trend as the predicted risk increased (Table 7, Figure 3), which confirmed that the model worked relatively well in the validation set.

**Table 7:** Readmission by predicted risk group in the validation set ^a^ p-values arise from either a Kruskal-Wallis or a χ2 goodness-of-fit test

	No 30-day readmission (n=1,093)	At least 1 30-day readmission (n=55)	P-value^a^
Predicted risk group, n (%)			<0.001>
Missing	1	0	
(0.000-0.032]	195 (17.9)	5 (2.5)	
(0.032-0.033]	407 (97.4)	11 (2.6)	
(0.033-0.050]	336 (95.7)	15 (4.3)	
(0.050-0.100]	125 (89.3)	15 (10.7)	
(0.100-0.200]	29 (76.3)	9 (23.7)	

**Figure 2:**
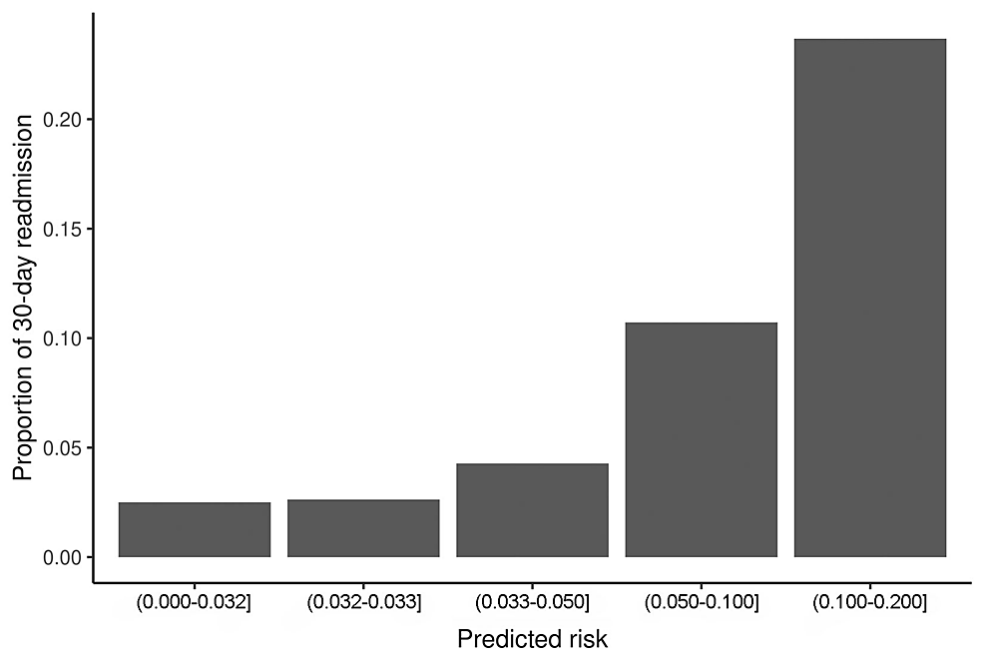
Predicted probability of 30-day readmission

## Discussion

The findings suggest that surgery type, LOS, and heart failure most significantly impacted 30-day readmission rates. At the researchers’ institution, the 30-day hospital readmissions rate after TKA and THA was 4.4%, and most of the sampled readmissions were surgery-related (68.2%). These findings align with similar studies reporting 30-day readmission rates and reasons for readmissions [1,7,16,17]. Patient variables found to be associated with a significant risk of readmissions included weight, BMI, heart failure, chronic obstructive pulmonary disease, dialysis, and a history of alcohol use or abuse. Further examination of weight and BMI indicated that being underweight (BMI<18.5) was also a significant characteristic related to hospital readmissions. Underweight patients, particularly if they were also undernourished, tended to experience adverse outcomes after orthopedic surgery [18]. This may have been because their bodies lacked the nutrients needed to heal after the procedures [19]. However, the current research on BMI and its association with surgical adverse effects is unclear. Ali and colleagues [18] suggest that high BMI was an independent predictor of readmission; however, Scully et al. [19] suggest that categorical BMI cannot be clearly delineated, but that overweight (BMI 25.0-29.9) and obese patients (BMI ≥30) suffer different adverse effects than patients who are underweight. Maurer et al. indicate that there is a relationship between BMI, degree of trauma, and hospital readmissions and that BMI should not be considered in isolation [16,20]. It is well established in the existing literature that comorbidities increase the risk for postoperative adverse effects. Heart failure and chronic obstructive pulmonary disease are strongly associated with postoperative adverse effects, with relative risk increasing 4.5 and 2.5, respectively [21-23]. This aligns with a previous study that suggested that trauma and patients with comorbidities such as myocardial infarction, diabetes, and hypertension also had increased readmissions rates [24]. Patients with cardiovascular disease may benefit from more intense preoperative cardiology input or closer monitoring postoperatively. Dialysis and alcohol use or abuse are also notable contributors [22,25]. When readmission occurred in our study population, the average LOS increased from 3.1 to 3.5 days.

The study also indicates that LOS and non-elective surgeries were significant contributors to hospital readmissions in our study group. Patients with longer LOS were significantly more likely to be readmitted. However, LOS could also be determined by preoperative comorbidities and, to a lesser extent, patient characteristics (e.g., age, sex, smoking status) [26]. Thus, this group could also benefit from physician-directed optimization prior to surgery. Patients who undergo non-elective total joint arthroplasty have inferior outcomes compared to those who have elective surgeries [27]. In our study, non-elective orthopedic surgeries accounted for 27.5% of all 30-day hospital readmissions. 

Despite legislative support for quality improvements, more must be done. During our study period, readmission rates in the US did not show significant improvement [26,28]. This underscores the concept that legislation alone is insufficient to significantly impact readmissions [1].

Studies of predictive models advocate for using them to aid decision-making that considers comorbidities, aftercare, and patient education and communication [6]. Advanced analytics and predictive models will provide surgeons with the ability to preoperatively assess patients who may or may not be good candidates for elective procedures, optimize care for those who require non-elective surgeries, and plan appropriately for patients who will need more preoperative or postoperative care [27]. Health care providers should integrate cost-effective measures, combining patient characteristics and risk factors. Surgeons may use such information to guide treatment decisions that optimize quality outcomes and provide patient-specific care in preoperative counseling, education, and clinical support. 

Predicting the future can be difficult. However, by preoperatively using common patient information, the study team believes that a suitably accurate model can be made to aid decision-making that optimizes patient outcomes. As this model was developed to predict 30-day hospital readmissions, it may not be useful for predicting mortality or morbidity outcomes. However, these outcomes are vital, and future studies should consider similar models to predict other outcomes of interest [28]. The interactions among variables and their total contributions to the models should also be considered. For example, researchers could examine the interactions between BMI, disease severity, and hospital readmissions or determine the extent to which LOS can be modified without sacrificing quality or access to care.

Although this study had the advantage of using a large database, its retrospective nature may have introduced selection bias. It is also possible that data may have been missing from electronic health records. Some of the potential predictors, such as smoking status and alcohol consumption, came from patient surveys, which were not as complete and accurate as the other data. This created difficulty in assessing the true association between these variables and 30-day readmission outcomes. We were also unable to identify whether readmissions occurred in other institutions outside of our network. Administrative data were used to classify the International Statistical Classification of Diseases, Tenth Revision, and all nonsurgical causes of readmission were assigned based on these codes. While coding is performed by trained personnel, it may not reflect the primary reason for readmission but rather another major concern treated during hospitalization. It is important to note that patients included in this study were cleared for surgery, so they were already screened for risk. This may have affected the prediction process. The literature largely supports the use of predictive models to aid surgeons in decision-making; however, we recognize that the variables in our study are not exclusive.

## Conclusions

Our research indicated that surgery type, length of stay, and heart failure most significantly impacted 30-day readmission rates. Once identified preoperatively, they can be adequately treated by physicians or specialists. By equipping medical professionals with added information, the authors aim to give them the ability to lessen readmission occurrence. This has practical implications for the clinical and economic effects of hospital and physician decisions.
